# Implications of Disrupted Circadian Rhythms on Pain

**DOI:** 10.20900/jpbs.20210014

**Published:** 2021-08-06

**Authors:** Jacob R. Bumgarner, Randy J. Nelson

**Affiliations:** Department of Neuroscience, Rockefeller Neuroscience Institute, West Virginia University, Morgantown, WV 26505, USA

**Keywords:** circadian rhythms, pain, circadian rhythm disruption

## Abstract

Pain is regulated by circadian rhythms. Daily fluctuations in pain thresholds are observed in health and disease. Disruptions to the circadian and pain systems may initiate a detrimental feedback loop between the two systems. The relationship between the pain and circadian systems is briefly reviewed to highlight a perspective on the need to consider disrupted circadian rhythms in the treatment of pain.

## CIRCADIAN RHYTHMS of PAIN SENSITIVITY

An essential component to health is the synchronization of behavioral and physiological processes to daily environmental cycles [1]. The synchronization of these processes occurs in the form of circadian rhythms, which are present in nearly every terrestrial organism. Circadian rhythms, endogenous biological clocks that cycle over the period of around 24 h, are synchronized to the external environment primarily by solar light cues. By coordinating and optimizing the timing of behavior and physiology in relation to the external day, circadian rhythms can improve individual fitness [2].

Numerous examples of circadian rhythms are commonplace: e.g., sleep, metabolism, body temperature fluctuation, immune function, and cognitive performance. But a lesser-considered circadian rhythm is pain sensitivity [3,4]. Indeed, pain sensitivity rhythms can be defined as true circadian rhythms as determined by the persistence of threshold variations in constant conditions [5,6]. Circadian rhythms in pain sensitivity were first noted in the late 20th century, but given the complexity of the distributed pain system, mechanistic insights into the underpinnings of these rhythms have been scarce. However, current evidence points to the descending pain modulatory system and spinal cord as the primary regulators of circadian rhythms of pain. Future research should continue to explicitly examine the role of individual rhythms in the nociceptive system and their contributions to daily variations in pain thresholds.

Circadian rhythms of pain thresholds likely serve some adaptive function. As humans and rodents appear to have the highest pain thresholds during their active phases [7–9], gated pain transmission while awake may facilitate improved focus on behavioral output, such as foraging or navigation, whereas facilitated pain transmission during the inactive phase may promote wound-healing behaviors.

## CIRCADIAN RHYTHM DISRUPTION and PAIN: A FEEDBACK LOOP

The functional connection between the circadian and pain systems makes the two susceptible to perturbations from one another. Disruption of one system can impact the other.

Emerging evidence from human research suggests that circadian rhythm disruption can negatively affect the function of the pain system. Circadian rhythm disruption occurs when external factors desynchronize the biological clocks of internal systems; light at night, shift work, and social jet lag are cues all common examples of circadian rhythm disruptors [10]. For example, night shift work has been associated with increased back pain [11,12] and increased sick leave due to lower back pain [13]. Night shift work is also associated with reduced heat [14] and cold pain thresholds [15]. Additionally, sleep deprivation has detrimental effects on pain processing [16]. Reduced heat, cold, and mechanical thresholds have all been observed following sleep deprivation, but the reported effects have been inconsistent [17].

**Figure 1.**
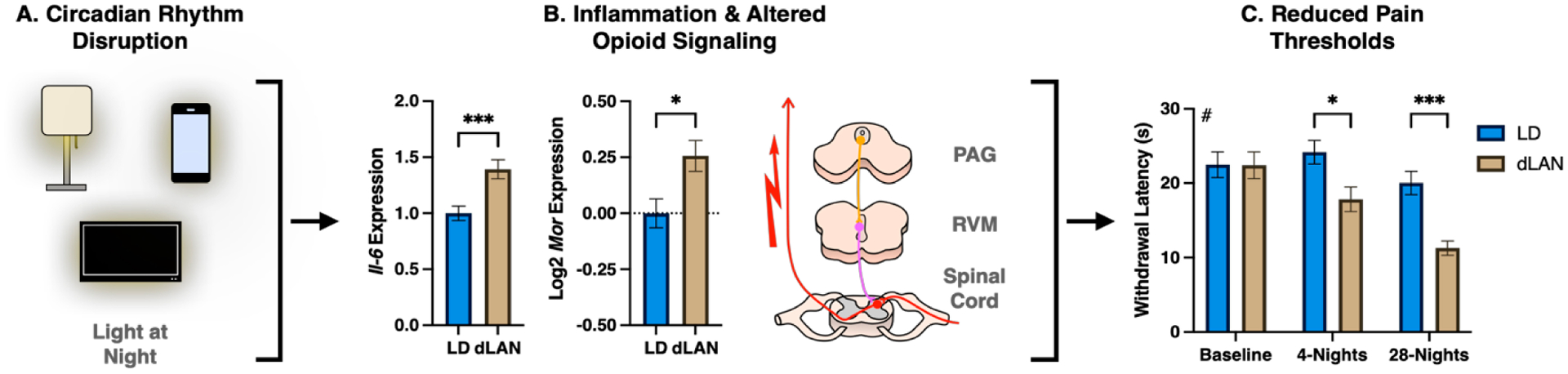
Circadian rhythm disruption alters pain thresholds. (**A**) Male CFW mice at 8-weeks of age were exposed to control light-dark cycles (14: 10 h light-dark; LD) or dim light at night (14:10 light-dim; 5-lux at night; dLAN) for four weeks. (**B**) Mice housed in dLAN for 28-nights exhibited heightened *Il-6* and *Mor* transcript expression in the medulla and periaqueductal gray, respectively. (**C**) Mice housed in dLAN for 4- and 28-nights exhibited cold hyperalgesia to a cold plate test at 0 ± 1 °C. Gene expression data were analyzed with unpaired 2-tailed *t*-tests. Behavioral data were analyzed using a repeated measures ANOVA. Data in the figure were modified with permission from Bumgarner et al., 2020 [20]. ^#^ —Main effect of lighting condition, * *p* < 0.05, *** *p* < 0.001.

In nonhuman animals, disruption of circadian rhythms can also reduce pain thresholds. For example, as with humans, sleep deprivation reduces pain thresholds in rodents [17]. Mouse models of chronic jet lag and mis-timed eating produce similar effects [18,19]. Another study demonstrated that exposure to a ubiquitous circadian rhythm disruptor—light at night—can reduce cold pain and mechanical thresholds in mice [20] (Figure 1).

The circadian system is susceptible to perturbation by pain. In several disorders and injuries associated with neuropathic pain, physiological and behavioral rhythms become disrupted. For example, humans with fibromyalgia have altered sleep-wake cycles [21], and although correlative, melatonin and cortisol rhythms are perturbed in humans with cervical spinal cord injuries [22]. Rodent studies have demonstrated similar effects of neuropathic pain and spinal cord injury on altered behavioral and physiological circadian rhythms [23,24].

Together, evidence demonstrating the reciprocal relationship between disrupted circadian rhythms and altered pain thresholds suggests the existence of a feedback loop between the pain and circadian systems. Circadian rhythm disruption heightens the sensitivity of the pain system, and circadian rhythms are either directly or indirectly disrupted in states of chronic pain or neuropathy. This feedback loop may be particularly detrimental in states of chronic circadian rhythm disruption, such as night shift work, or in the presence of serious chronic or neuropathic pain conditions, such as fibromyalgia. Future work examining the interactions of circadian rhythm disruption and other neuropathic pain states, such as diabetic peripheral neuropathy or chemotherapy-induced peripheral neuropathy may be insightful for improved therapeutic interventions.

## Data Availability

No data were generated for this article.

## CLINICAL IMPLICATIONS

Pain therapy can benefit from increased consideration of the relationship between circadian rhythms and pain responses. Circadian rhythms are already considered in phototherapeutic [25,26] or pharmacological treatment of pain conditions [27,28]. However, the relationship between disrupted circadian rhythms and exacerbated pain in health or disease has been underappreciated in the clinic. Lifestyle modifications necessary to eliminate or reduce circadian rhythm disruption are low risk and often non-invasive. Yet, the therapeutic potential of these modifications may be significant, given the present need for additional non-pharmacological therapeutic options for pain [29]. Improving patient quality of life and reducing pain symptoms may be as simple as instructing patients to minimize circadian rhythm disruption by maintaining regular sleep schedules, eating regularly timed meals, eliminating exposure to light at night, and maximizing exposure to bright light during the day.
